# A 12-year retrospective evaluation of anal pre-cancerous lesions and cancer in people living with HIV-1 infection in the Southeastern U.S

**DOI:** 10.1186/s13027-021-00354-7

**Published:** 2021-02-17

**Authors:** Yuanfan Ye, Greer A. Burkholder, Amrita Mukherjee, Daniel Chu, Anju Bansal, Staci L. Sudenga, Anna Junkins, Sameer Al Diffalha, Michael S. Saag, Sadeep Shrestha

**Affiliations:** 1 Department of Epidemiology, School of Public Health, University of Alabama at Birmingham, Birmingham, AL 35294 USA; 2 Department of Medicine, Division of Infectious Diseases, School of Medicine, University of Alabama at Birmingham, Birmingham, AL 35294 USA; 3 Department of Surgery, School of Medicine, University of Alabama at Birmingham, Birmingham, AL 35294 USA; 4 Department of Medicine, Vanderbilt University, Nashville, TN 37203 USA; 5 Department of Pathology, School of Medicine, University of Alabama at Birmingham, Birmingham, AL 35294 USA

**Keywords:** Anal cancer, Anal HSIL, PLWH, Anal screening, HIV-positive MSM

## Abstract

**Background:**

Anal cancer is rare in the general population in both genders in the US, but an increased incidence of anal cáncer (AC) has been reported among people living with HIV-1 infection (PLWH) and little is known among the population in South US.

**Methods:**

In a retrospective study design, electronic health records from 2006 to 2018 were reviewed in a HIV clinical cohort at the University of Alabama at Birmingham. Associations of demographic, sociodemographic, and HIV-clinical indicators were examined in univariate analyses between high-grade squamous intraepithelial lesions (HSIL) and AC cases and condition-free individuals. Factors for anal/rectal cytology screening tests among PLWH were also assessed over time. Ages at onset of anal cancer were compared with the general US population reported by the National Surveillance, Epidemiology, and End Results Program.

**Results:**

A total of 79 anal HSIL (96% men) and 43 cancer (100% men) patients were observed along with 4367 HSIL/cancer-free patients (75.9% men). HSIL (*P* < 0.0001) and AC (0.0001 < *P* < 0.01) were associated with being men who have sex with men (MSM). An incidence of 258 per 100,000 person-year was observed among this clinical cohort of PLWH. PLWH who were 45–54 years appeared to be at highest risk of AC (58.1%), as compared to those 55–64 years in the general population. Overall, 79% of PLWH anal cancers were diagnosed among those under 55 years (vs 39.5% in general population) indicating early onset of AC. In total 29.1% of HSIL and 44.2% of AC patients had not received an anal/rectal cytology examination 1 year prior to diagnosis.

**Conclusion:**

AC incidence among HIV-infected men was 161 times higher than general population with an earlier age of onset/diagnosis. Many patients with AC had missed screening opportunities that could potentially have captured neoplasia in pre-cancerous stages. AC-related screening guidelines need to be integrated into routine clinical care, especially among PLWH at highest risk such as MSM and those with lower CD4 counts.

## Introduction

Anal cancer (AC) is rare in the general United States (US) population, with an estimate of 8,300 new diagnoses, 5,530 in women and 2,770 in men, reported in 2019 [1]. Lifetime risk of developing anal cancer is about 1 in 500, with a higher risk in women than men [2, 3]. However, the incidence of AC has been increasing over recent years in some high-risk populations [4], particularly among people living with human immunodeficiency virus (PLWH) [5]. Individuals co-infected with High-risk Human Papillomavirus (HR-HPV) and HIV, and men who have sex with men (MSM) have been identified as major factors that increase the risk of anal cancer [6–8].

It is known that 91% of ACs are directly linked with persistent HR-HPV infection [4, 9–11]. HPV infection does not clear as spontaneously among immunocompromised individuals, resulting in a higher risk of developing physical symptoms and possibly progressing to neoplasia in the infected tracts [12]. A systematic review by Machalek et al. published in Lancet Oncology in 2012 estimated a pooled anal cancer incidence of 77.8 per 100,000 person-years (PY) among HIV-infected MSM, in the post highly active antiretroviral therapy (HAART) era [13]. By contrast, the incidence of anal cancer is 1.9 per 100,000 (PY) in the general US population [14]. The same meta-analysis also reported that incidence of neoplastic lesions, an immediate precursor of squamous cell carcinoma (SCC) in the anal tract, varied between 8.5–15.4% per year among HIV-infected MSM [13]. However, in recent years, incidence of AC has been trending up, warranting further research on demographic distribution, socio-behavioral risk factors, and screening strategies in highly vulnerable target populations, such as PLWH.

AC is not categorized as an acquired immunodeficiency syndrome (AIDS)-defining malignancy, and overall, it is rare in the general population. Thus, it is generally overlooked. Given that over 52% of HIV in the US is diagnosed in the Southern US [15], it is important to study this high-risk population. Thus, we conducted a retrospective study within an academic clinic in this region to examine the epidemiology and clinical practices to screen and diagnose anal cancer and its precancerous lesions, specifically high-grade squamous intraepithelial lesions (*HSIL*).

## Methods

### Study setting and population

A retrospective study nested within an ongoing clinical cohort in the HIV Clinic (known as the 1917 Clinic) at the University of Alabama at Birmingham (UAB) was conducted using electronic health records (EHR) between January 1st, 2006 and March 30th, 2018. The UAB 1917 Clinic is the largest HIV care facility in the state of Alabama with an extensive regional catchment and referral network. The prospective clinic cohort has collected data on more than 7000 patients including self-reported sociodemographic, behavioral and psychological characteristics, co-morbidities, medications, vital signs, and laboratory results since the cohort establishment in 1992 [16]. Currently, more than 3500 patients receive their routine HIV care from the clinic, representing 30% of all PLWH in the state. In addition to routine HIV care, local PLWH also receive primary care, cancer screening referrals, and other medical needs through the 1917 Clinic.

The present study was approved by the UAB Institutional Review Board for human studies.

### Eligibility criteria

Patients included in the study attended the 1917 Clinic at least twice for routine HIV-care during the study period and were at least 18 years old at HIV diagnosis. Medical charts were reviewed and verified for all cases to confirm the diagnoses of anal HSIL and cancer. Only cases diagnosed during the study period are identified as incident cases to fit the epidemiological definition. Anal dysplasia-related diagnoses 1 year prior to the study enrollment were also abstracted from EHR. PLWH with such documented anal HSIL and cancer were excluded from the study to avoid over-counting incident cases. Data were obtained by query of the cohort’s electronic database using MS SQL Server 2008.

### Statistical analyses

Univariate analyses were performed to compare demographic, behavioral, and clinical characteristics between HSIL and AC cases to condition-free patients. Categorical variables were reported as frequencies with percentages and continuous variables as median interquartile range (IQR). Race, gender, self-reported sexual orientation, education, marital status, and employment status, ages at HIV diagnoses and study enrollment, receipt of standard anal/rectal cytology test (anal Pap), and CD4 T-cell count and viral load (VL) at the anal HSIL or cancer presentation were assessed. In order to evaluate clinical awareness of anal cancer screening, demographic and sexual risk factors were compared between patients who received at least one standard anal/rectal cytology test and those who were never tested during the study period. Chi-squared tests were used to assess the differences of categorical variables and T-tests were used for the comparison of continuous variables. Fisher’s exact test was used when the cell counts were small (< 5).

Trends of incidence of HSIL and AC were analyzed for every consecutive 4-year period (years 1–4, 5–8, 9–12, 13–16) since the patient’s initial visit to the 1917 Clinic, which could be dated back to 1999 based on available administrative system data. The follow-up ended at the last routine clinical visit for patients without anal HSIL or cancer and at the onset date of diagnosis for patients with HSIL or cancer. Cumulative risk was estimated as the incident anal HSIL or cancer divided by the total condition-free (without anal HSIL/cancer) population when they entered each 4-yr period. Individuals with confirmed cases were excluded from the cohort after their case period. The trend of cumulative risk was estimated using a generalized log-linear regression model for each period. Log-transformed number of people at risk during each 4-year period was set as an offset for the model. There were very few individuals who were followed for 17–20 years, so this period was underpowered for analyses.

The age distribution of anal cancer in the present clinic cohort was compared to the general US population using the Surveillance, Epidemiology, and End Results (SEER) Program data from the National Cancer Institute [17]. Percentages of anal cancer in each standard age group were plotted side by side with the SEER data.

## Results

**Fig. 1:**
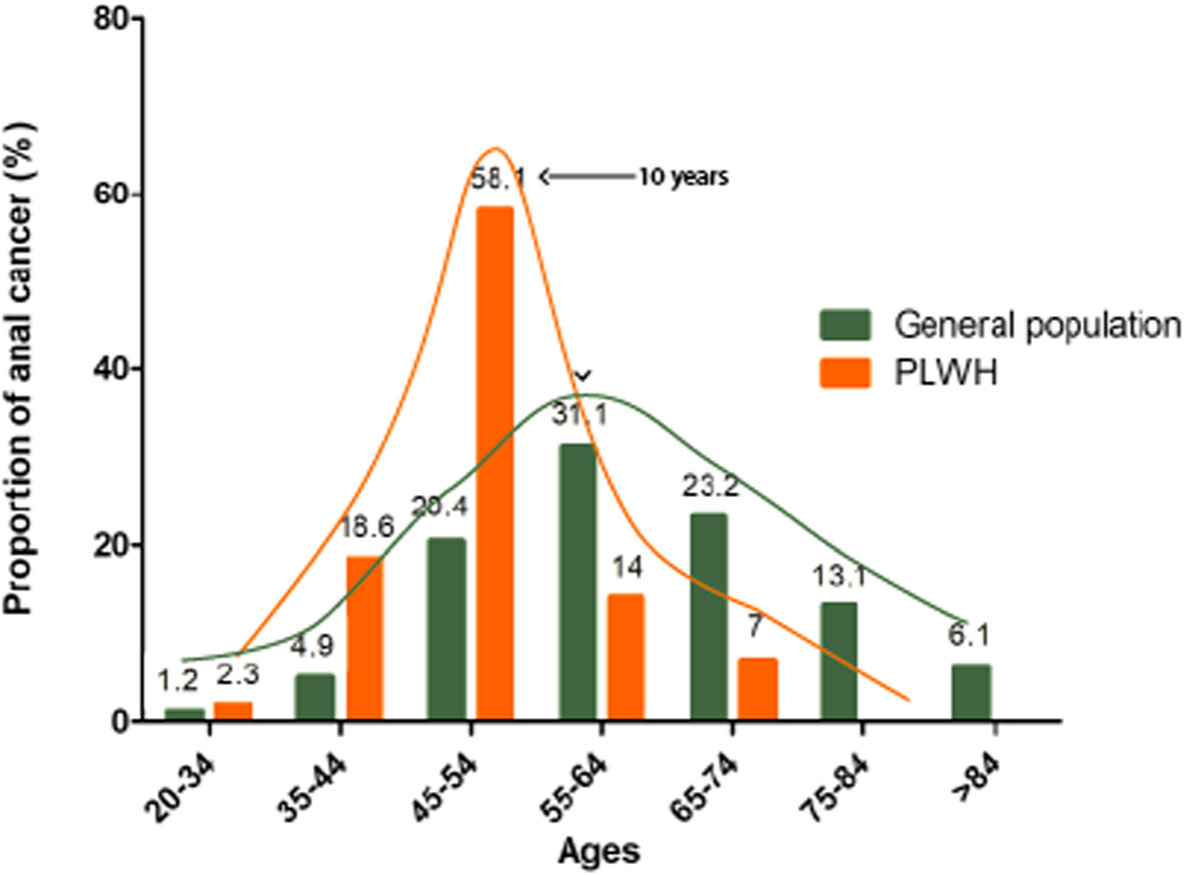
Ages at anal cancer diagnosis among PLWH compared to the general US population from the SEER. Percentage of each age group is given

There were 79 anal HSIL and 43 cancer cases observed among 4482 patients during the 12-year follow-up period. Only three HSIL cases were observed in women (4.0%), and no AC cases were detected in women (Table 1). The gender distribution was significantly different between HSIL and AC patients compared to the condition-free patients (*P* < 0.0001 for each). Most male HSIL (93.5%) as well as AC (82.9%) patients self-reported as MSM (Table 1). Median age at HSIL diagnoses was 7.6 years younger than AC diagnoses (0.0001 < *P* < 0.05). Most AC diagnoses were in the 45–54 year age group (58.1%) (Fig. 1). While only 26.5% of ACs occurred among individuals younger than 55 years in the general population, we observed that 79% of anal cancers occurred in the same age range among PLWH (Fig. 1). About 62.8% AC patients had nadir CD4 counts less than 200 copies/mL, whereas only 38.9% condition-free patients fell in the group (0.0001 < *P* < 0.05). More HSIL (22.8%, 0.0001 < *P* < 0.01) and AC (32.6%, *P* < 0.0001) patients had median CD4 counts less than 200 copies/mL compared to the the condition-free group (14.0%). A slightly higher proportion of individuals with HSIL had received anal/rectal cytology tests 1 year prior to their diagnosis than the AC patients (51% vs 33%, *P* < 0.0001), but the difference was not statistically different (Table 1). A higher proportion AC patients reported being married or with partners compared to AC and HSIL free patients (20.9% vs 14.3%, 0.0001 < *P* < 0.05), whereas most of HSIL patients reported being single compared to AC and HSIL free patients (93.6% vs. 73.4%, 0.0001 < *P* < 0.05) (Table 1). More than double AC patients claimed disability compared to HSIL and AC free patients (55.3% vs. 27.1%, 0.0001 < *P* < 0.05) (Table 1).

**Table 1:** Social-behavioral and clinical characteristics of anal HSIL and cancer cases compared with non-HSIL or cancer subjects (*N* = 112 individuals) from the 1917 clinic

	Anal HSIL (***N*** = 79)	Anal cancer (***N*** = 43)	Anal HSIL and cancer free (***N*** = 4367)
**Race**			
Blacks	42 (53.2)	18 (41.9)^‡^	2618 (60.0)
Whites	36 (45.6)	24 (55.8)^‡^	1575 (36.0)
Others	1 (1.2.)	1 (2.3) ^‡^	174 (4.0)
**Gender**			
Men	76 (96.0)*	43 (100)*	3315 (75.9)
Women	3 (4.0)*	0 (0)*	1028 (23.5)
Transgender individuals	0 (0)*	0 (0)	24 (0.50)
**Self-reported sexual risks**			
Heterosexual	5 (6.5)*	7 (17.1)^‡^	1826 (43.5)
MSM	72 (93.5)*	34 (82.9)^‡^	2360 (56.2)
Others	0 (0)*	0 (0) ^‡^	11 (0.30)
**Median age at HIV diagnoses (years)**	28.8 (24.6–37.7)^‡^	31.7 (25.6–38.8)	32.9 (26.4–54.0)
**Median age at study enrollment (years)**	38.4 (27.3–46.7)	44.9 (40.7–50.9)^‡^	40.9 (31.8–59.1)
**Median age at anal HSIL, cancer, or last clinical visit (years)**^**‡**^	43.8 (31.8–54.2)	51.4 (46.9–54.6)	46.1 (36.2–54.6)
**Nadir CD4 count (cells/μL)**			
< 200	36 (45.6)	27 (62.8)^‡^	1699 (38.9)
200–500	28 (35.4)	11 (25.6)^‡^	1819 (41.7)
> 500	15 (19.0)	5 (11.6)^‡^	849 (19.4)
**Median CD4 count (cells/μL)**			
< 200	18 (22.8)^‡^	14 (32.6)*	612 (14.0)
200–500	28 (35.4)^‡^	19 (44.2)*	1606 (36.8)
> 500	33 (41.8)^‡^	10 (23.3)*	2149 (49.2)
**Highest VL (copies/mL)**			
< 50 (undetectable)	13 (16.5)	6 (14.0)	430 (9.9)
> 50 (detectable)	66 (83.5)	37 (86.0)	393 (90.1)
**Median VL (copies/mL)**			
< 50 (undetectable)	44 (55.7)	21 (48.8)	2641 (60.5)
> 50 (detectable)	35 (44.3)	22 (51.2)	1726 (39.5)
**Ever received anal/rectal Pap 1 year before diagnosis**			
Yes	40 (50.6)	14 (32.6)	
No	39 (49.4)	29 (67.4) *	
**Marital status**	*N* = 78	*N* = 43	*N* = 4240
Married/life partner	3 (3.9)^‡^	9 (20.9)^‡^	609 (14.3)
Divorced/separated	2 (2.5)^‡^	3 (7.0)^‡^	395 (9.3)
Single	73 (93.6)^‡^	29 (67.4)^‡^	3111 (73.4)
Widowed	0 (0)^‡^	2 (4.7)^‡^	125 (3.0)
**Employment status**	*N* = 73	*N* = 38	*N* = 3197
Unemployed	13 (17.8)	5 (13.2)^‡^	660 (20.7)
Disabled	23 (31.5)	21 (55.3)^‡^	866 (27.1)
Employed	31 (42.5)	9 (23.7)^‡^	1417 (44.3)
Retired	4 (5.5)	2 (5.3)^‡^	208 (6.5)
Student	2 (2.7)	1 (2.5)^‡^	46 (1.4)

*HSIL* high-grade squamous intraepithelial lesion, *MSM* men who have sex with men, *HIV* human immunodeficiency virus, *VL* viral load

*: *P* < 0.0001; ^‡^:0.0001 < *P* < 0.05

Among the entire cohort of 4482 patients, 1648 (36.8%) received at least one anal/cytology test during the study time. More men received the tests (47.0%) compared to very few women overall (1.5%) (*P* < 0.0001). There were 46.8% Whites and 31.3% Blacks receiving anal pap tests (*P* < 0.0001), compared with only 27.3% of other races. Based on self-reported sexual orientation, only 8.4% heterosexual men received the tests while it was higher among MSM (62%) (*P* < 0.0001). Among individuals who developed HSIL, 29.1% had never received the screening tests during the study periods. Likewise, 44.2% of AC patients also had never received a screening test (*P* < 0.0001) (Table 2).

**Table 2:** Patients receiving anal/rectal pap tests between January 2006–March 2018

	Ever received (***N*** = 1648)	Never received (***N*** = 2834)	***P***-value
**Gender**			
Men	1611 (47.0)	1816 (53.0)	< 0.0001
Women	16 (1.5)	1015 (98.5)	
Transgender M-F	21 (87.5)	3 (12.5)	
**Race**			
Black	836 (31.3)	1839 (68.7)	< 0.0001
White	764 (46.8)	867 (53.2)	
Others	48 (27.3)	128 (72.7)	
**Self-reported sexual risk factors**	*N* = 1591	*N* = 2683	
MSM	1504 (61.7)	932 (38.3)	< 0.0001
Heterosexual men	72 (8.4)	781 (91.6)	
Heterosexual women	15 (1.5)	970 (98.5)	
**Anal HSIL and cancer**			
HSIL	56 (70.9)	23 (29.1)	< 0.0001
Cancer	19 (55.8)	24 (44.2)	
**Race- sexual behavior**	*N* = 1591	*N* = 2683	
Black MSM	732 (62.3)	442 (37.7)	< 0.0001
Black heterosexual men	51 (8.1)	578 (91.1)	
Black heterosexual women	10 (1.4)	731 (98.6)	
White MSM	729 (62.0)	446 (38.0)	
White heterosexual men	19 (10.6)	161 (89.4)	
White heterosexual women	5 (2.3)	213 (97.7)	
Other race MSM	43 (49.4)	44 (50.6)	
Other race heterosexual men	2 (4.6)	42 (95.4)	
Other race heterosexual women	0 (0)	26 (100)	

*M-F* male to female, *MSM* men who have sex with men, *HSIL* high-grade squamous intraepithelial lesion

**Fig. 2:**
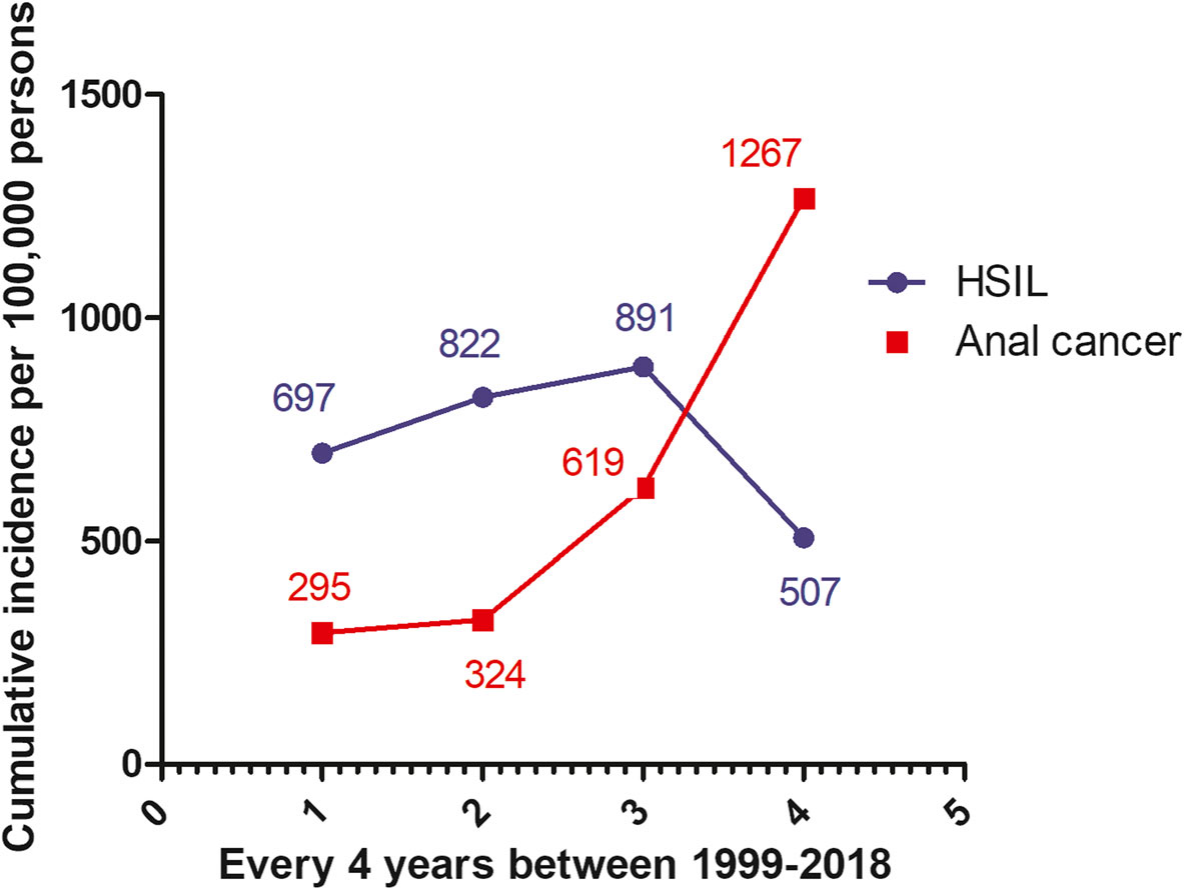
Cumulative risk of anal HSIL and cancer every 4 years (0–4, 5–8, 9–12, 13–16 years) since patient initial clinical visit dates. The current cohort began in 1999 and ended in early 2018. The last period (17–20 years) was excluded due to an incomplete 4-year follow-up. Diagnoses of anal HSIL declined while cancer cases increased during the follow-up

The trends of incidence over the four defined analysis periods for anal HSIL and cancer were in opposite directions. Cumulative incidence of HSIL started to decline after year 12 (3rd period) since patients’ initial clinical visit dates, whereas the incidence of anal cancer increased constantly overtime (Fig. 2). The cumulative risk of AC increased constantly from 295 to 1267 per 100,000 persons, whereas the cumulative HSIL risk decreased from 697 to 507 per 100,000 persons (Fig. 2).

## Discussion

Our study describes the relevant sociodemographic and clinical characteristics of PLWH diagnosed with anal HSIL and cancer in an urban HIV clinical in the Southeastern U.S. The overall incidence rates of HSIL in our clinical cohort were 338 and 194 per 100,000 person-years among men and women, respectively, with a rate of 258 AC cases per 100,000 men-years (no women presented with AC in the study) during the 12-year follow-up [18]. By contrast, data from SEER suggest that the incidence of AC in the general US population is 1.6 cases per 100,000 person-years in men and 2.2 cases per 100,000 person-years in women [14]. This is an alarming 161-fold higher incidence in HIV-infected men in the study compared with men in the general population, and 102-fold increase compared to both genders in the general population. HIV-infected men accounted for 96.0% of HSIL and 100% of AC cases in this study, while the study cohort consisted of 23% women. Recent studies among women living with HIV in US have reported the prevalence of anal HSIL to be 27% [19]. Our previous findings indicated that HIV-infected men had 7.9 times higher risk of incident anal HSIL (432 per 100,000 person-years), compared with HIV-infected women (55 per 100,000 person-years) [18]. Of note, during this study period, 17 out of 79 patients (21.5%) regressed to low grade squamous cell lesions (LSIL). All 43 AC cases occurred in men; and resulted in an incidence rate of 258 per 100,000 men-years [18].

Our study follow-up was between 2006 and 2018, during which combined antiretroviral therapy (cART) had been widely prescribed, specifically since 2012 in the US. The Adult and Adolescent Spectrum of the HIV Disease Project and HIV Outpatient Study from 1992 to 2003 reported an increase of incidence of AC from 19 to 78.2 per 100,000 person-years and suggested that increase was related to the longer exposure period to HPV infection [20]. PLWH were able to live a nearly normal life span during the study time because of cART. In 1996, the life expectancy for a 20-year-old person with HIV was only 39 years. By contrast, by 2011, the life expectancy had risen to about 70 years old in the general population [21]. AC usually develops among people in their early 60s, and diagnoses are uncommon in people younger than 35 years old [1]. Thus, during the early years of the HIV epidemic, it would have been rare to observe anal cancer among PLWH, as they typically did not survive to their 60s. The median age at AC diagnosis in the general US population is 62 years with most frequent diagnoses made between 55 and 64 years (31.1%) (Fig. 1). The median age in our study population was 10 years younger with majority in the 45–54 years group (58.1%). Approximately 79% of anal cancers were observed in individuals under 54 years in the current study vs in the general population (Fig. 1). Of note, there were very few patients > 74 years in the study cohort; thus, we focused more on the young and middle-aged HIV population. The shorter life expectancy of HIV individuals during the early HIV epidemic did not allow HPV-infection to be sufficiently persistent to develop neoplasia.

We were not able to assess some of the SES variables due to unavailable data. For example, only 44% of HSIL, 26% of AC and 40% of HSIL/AC free individuals self-reported education and none of the groups were statistically different in these subgroups (data not shown). There were statistically significant differences with marital status distribution among the three groups (Table 1). Overall, 92% of HSIL, 88% of AC and 73% of HSIL/AC free individuals self-reported employment status. HSIL/AC free patients were more unemployed and higher proportion of both HSIL and AC patients reported disability. However, it is premature to make any interpretation of this observation due to small sample size in this subset analysis (Table 1).

In general, prolonged lifespan with the universal cART coverage effectively restores the impaired immune systems and should have reduced the risk of having persistent HPV-infection. In this context, we would have expected lower AC risk at earlier age [22], but higher AC occurrence at older age [23]. However, a recent study suggested that the high incidence of AC in young PLWH could potentially be due to early and profound immunosuppression coupled with suboptimal immune recovery [24]. While the treatment has been effective overall during the cART era, the present study also indicated that patients with nadir CD4 < 200 (cells/μL) accounted for 45.6 and 62.8% of anal HSIL and cancer, respectively (Table 1). Only 23.3% of AC patients had median CD4 counts ≥500 (cells/μL) (Table 1) suggesting that a large proportion did not have optimal immune status prior to cancer diagnosis. Lower CD4 count (≤200 cells/μL) is known to be a key factor in anal HSIL and cancer development [19, 25]. While CD4+ could be a proxy for HIV status or treatment effect, there are no standard guidelines for anal HSIL treatment so that could not be assessed in the current study.

While at least half of HSIL patients in our clinical cohort received at least one anal/rectal cytology test, a year prior to the onset of HSIL diagnosis, over 67% of AC patients did not and the missed opportunity to screen the carcinoma in the precancerous stages is a public health concern. There were higher numbers of anal HSIL diagnoses in the earlier 3 periods of the 4 four-year defined periods, but a decrease was observed after year 12 (Fig. 2). By contrast, AC incidence remained low in the first 8 years of attendance at the HIV clinic and increased significantly after year 8, suggesting that there were missed opportunities of screening at pre-cancerous stages in addition to prolonged immune suppression. These data suggest that from a clinical standpoint, anal cancer prevention strategies could be considered to be integrated into routine clinical care, especially among PLWH [14]. Some studies have shown the effectiveness of anal cancer screening guidelines in targeted individuals, such as MSM and those with a history of condylomata, and women with cervical or vulvar dysplasia [2]. However, many inconsistencies have been observed in screening processes, and post-screening recommendations are not standardized. Sexual orientations are self-reported and result in an under-reported number of MSM due to societal stigma [26]. In our study, self-reported MSM represented most of the HSIL (93.5%) and AC (82.9%) cases; however it could miss other high-risk individuals who may not identify them as MSM in different settings where stigma against MSM exist. The American Cancer Society recommends an annual anal/rectal cytology test for the high-risk population but no specific screening schedule or age-group has been set [2]. Although, patients diagnosed with HSIL can ideally have lesions surgically removed, such invasive excisions are generally only performed in those with severe or extensive HSIL lesions [27–29], which remains subjective without consistent guidelines based on research. While several guidelines have been developed, whether and how to integrate an anal cancer-related screening program within a routine patient care still remain debatable [30]. Further, the current study did not examine the administration of high resolution anoscopy (HRA) because during the early time of the study follow-up, HRA was not commonly provided. Evaluating the results from HRA in this 12+ year follow-up would have been biased.

Cervical cancer is an AIDS-defining condition, screening and treatment related costs are covered by the Ryan White Program in Alabama, resulted in a reduction in incidence cases [31]. Although not fully known, it is possible that routine cervical cancer screening may potentially benefit women from progression of all other HPV-induced anogenital tract malignancies. Overall, non-AIDS-defining malignancies (NADM), specifically anal cancer are increasing in HIV-positive individuals [32, 33]. One prior study reported the prevalence of NADM was 10 times higher in patients with < 50 cells/μL CD4 counts than those with > 500 cells/μL [34]. It takes 15 to 20 years for cervical cancer to develop from untreated dysplasia in women with normal immune systems. However, it may take only 5 to 10 years for HIV-infected women [35]. Although we were limited with follow-up data of HSIL progression to cancer in the current study, there seems to be 7.6-year difference in median age between diagnosis of anal HSIL and cancer in our study population (Table 1). Another limitation of the study is that we used clinical diagnoses with no detailed laboratory information. For example, reflex testing for the presence of HR-HPV is not routinely performed on anal/rectal cytology, and therefore we cannot determine cases of HSIL and AC, which were associated with HPV versus another etiology or if the infection occurred before or after the occurrence of HSIL/AC. This study is from one clinic in the southeastern U.S., results may not be generalizable to other sites or geographic locations. However, the alarming rate of AC found in our study needs to be carefully monitored as it has potential to be an epidemic problem in this targeted population in the region.

## Conclusions

The premalignant and malignant lesions were mostly observed in young MSM with a much early age of diagnosis. Over 50% of the HSIL patients had received anal/rectal cytology tests within a year prior to the onset of HSIL, but only about 33% of AC patients had been tested during the same time window. Key HIV-related prognostic indicators such as nadir CD4 count and median VL were important with onset of anal HSIL and cancer diagnoses.

## Data Availability

All data can be obtained with formal request from the 1917 clinic Research and Informatics Service Center (RISC).

## Abbreviations

AC: Anal Cancer
cART: Combined AntiRetroviral Therapy
HAART: Highly Active AntiRetroviral Therapy
HIV: Human Immunodeficiency Virus
HPV: Human Papillomavirus
HSIL: High-grade squamous Intraepithelial Lesions
MSM: Men having Sex with Men
NADM: Non-AIDS-Defining Malignancies
PLWH: People Living with HIV
SEER: Surveillance, Epidemiology, and End Results
SCC: Squamous Cell Carcinoma
